# Development and Implementation of Strong Foundations, a Digitally Delivered Fall Prevention Program: Usability and Feasibility Pilot Exercise Cohort Study

**DOI:** 10.2196/67406

**Published:** 2025-02-28

**Authors:** Ryan Moran, David Wing, Hope Davey, Hava Barkai, Jeanne Nichols

**Affiliations:** 1 Exercise and Physical Activity Resource Center (EPARC), University of California San Diego, 9500 Gilman Dr. La Jolla, San Diego, CA, 92093, United States, 1 (858) 534-9315; 2 Department of Medicine, University of California San Diego, San Diego, CA, United States

**Keywords:** digital health, fall prevention, fall risk, older adults, geriatrics, system usability scale, Strong Foundations, feasibility, public health, user acceptance, exercise, usability, digital technology, mobile phone

## Abstract

**Background:**

Falls remain a major public health problem and a significant cause of preventable injury. Maintaining strength and balance by staying active can prevent falls in older adults, and public health advocates support referral to community exercise programs. Given the growth in use and acceptance of technological interfaces, there remains an interest in understanding the role of a synchronous exercise program designed to improve strength, postural alignment, and balance specifically designed to be delivered in a digital environment with respect to usability and feasibility.

**Objective:**

This study aims to design and implement a synchronously delivered digital fall prevention program to adults aged 60 years and older, to understand the usability, feasibility, and attendance.

**Methods:**

The “Strong Foundations” program, a 12-week, live, digitally delivered fall-prevention exercise program was informed from different existing in-person exercises and piloted to older adults who were considered a low fall risk by scores of 4 or less from the Centers for Disease Control and Prevention’s (CDC’s) Stopping Elderly Accidents and Deaths Initiative (STEADI) Staying Independent questionnaire. The System Usability Scale (SUS) measured usability and feasibility at the completion of this program, and digital measures of age-related function (timed up and go [TUG] and 30-second chair stand [30 CS]) were collected pre- and postintervention. Data were collected in 2021.

**Results:**

A total of 39 older adults were recruited and 38 completed the 12-week program with an average age of 72 years. The average SUS was 80.6, with an 85% attendance rate and an 8.5 (out of 10) self-reported satisfaction score. Digitally collected TUG and 30 CS statistically improved pre- and postintervention by 9% and 24%, respectively; by week 12, 64% (23/36) of participants improved in the timed up and go and 91% (32/35) improved the chair stands.

**Conclusion:**

There was excellent usability and acceptability for Strong Foundations, a novel fall-prevention program designed to be delivered digitally and promising improvement of objective measures of fall risk.

## Introduction

Fall-related injuries among older adults are a significant and growing public health concern. Over one-quarter of community-residing men and women 65 years of age and older fall annually [1,2]. Risk is compounded over time as a single fall predicts recurrent falls [3-7], and fall-related mortality has increased over 30% just between 2007 and 2016 [8]. Not only do falls represent a major cause of accidental death and mobility-related disability among older adults, but they also result in psychological distress, loss of independence, and a decrease in quality of life [9,10]. Despite the existence of various interventions and policies aimed at reducing fall risk in this population, the incidence of falls remains alarmingly high.

While fall risk is multifactorial, identification of risk factors, as well as referral to and participation in appropriate fall-risk reduction programs are established as an effective, evidence-based approach to reduce fall risk [2,11]. Specifically, targeted strength and balance exercises have consistently been shown to improve fall risk, and accordingly, the Centers for Disease Control and Prevention (CDC) has outlined an evidence-based clinical approach to identify those at risk for falls to help assess known risk factors and refer for community-based fall-prevention programs [12,13]. The public health use of this approach is further supported by the United States Preventive Services Task Force (USPSTF) “Grade B” recommendation to refer older adults for fall prevention exercise programs [14,15]. However, despite these resources and recommendations, clinical identification and referrals have been insufficient [16]; only approximately one-third of older adults report being asked about fall-risk by their health care providers, and similarly, only around a third of those who fall report discussing their fall and fall circumstances with their health care provider [17,18]. At the population level, conservative estimates suggest far fewer than 50% older adults meet recommended physical activity levels [19]. These findings mirror the growing epidemiological evidence of increased rates of falls in older adults. While some existing programs that improve fall risk and activity for older adults, including EnhanceFitness PA and Silver Sneakers may be available to some older adults with certain insurance plans at no additional costs [20], others may face substantial fees; furthermore, many eligible seniors do not use these programs, citing several barriers. Such barriers include general dislike of gyms and a lack of awareness or instructor suitability and generalizability, problems accessing appropriate classes, concerns about bad weather and driving, and perceptions about being “old” in these environments [21]. These issues are even greater among older adults residing in areas and communities that have fewer facilities for guided physical activity, more limited transportation infrastructure and less exercise expertise [22]. Finally, there remain geographic and demographic disparities related to falls, including higher rates in rural areas [2], highlighting the interest and need for different approaches to address this public health and clinically relevant problem in older adults in novel ways that increase reach and are safe and effective.

Appropriate technologically driven approaches have promise to increase range and dissemination of fall prevention resources, including making them easier and safer to access [23]. Furthermore, digitally delivered programs that involve appropriately tailored exercises offer the opportunity to balance risks and benefits during times of social distancing, while improving dissemination and community uptake, and possibly improve fall risk [24]. While recent attention has highlighted concerns with broadband access in older adult populations, especially with attention to disparities and equity [25], digital interventions showcase the promise as those aged 65 years and older still represent the fasting growing demographic in technological uptake [26,27]. Thus, given the trend of increasingly ubiquitous access to smart-enabled technology in older adults, and gaining acceptance in this population [26], there is great potential to improve equitable access to high-quality exercise-based fall prevention resources by decreasing geographic boundaries—not only immediately, but increasingly in the future. Alternative approaches, including prerecorded, online, and asynchronous delivery, are an additional consideration but lack the ability for real-time guidance, which is possible with synchronous real-time delivery, and therefore offer the opportunity to provide guidance with risk mitigation and personalization when appropriate for participants. Therefore, approaches that leverage technology to improve access to fall-risk reduction exercise programs are imperative, and growing data suggest digitally formatted delivery may be feasible [28].

Zoom (Zoom Video Communications) is a user-friendly, web-based service that provides digital video-based connections between multiple users; Zoom-for-Telehealth is a HIPAA-compliant encrypted service used for clinically delivered care and permits conferencing between individuals. Zoom is readily available online, free to download, and supports a one-to-many viewing platform such that it can be set up so that only the host can see individual participants at a given time [29]. This application was selected based on the ubiquitous nature and availability to host professionally through our institution. The nature of this digital platform lends interest in understanding how fall-prevention curricula may be developed and delivered to older adults; therefore, we developed a novel digital platform, Strong Foundations*,* designed to leverage the advantages of digital technology with evidence-based fall-prevention strategies, along with real-time feedback to users based on appropriate or inappropriate practice, optimizing gains for a larger group and highlighting core exercises. This program includes features to promote adherence, engagement, and social connectivity enabled by real-time interaction, all important to promote efficacy and subsequent improvement in gains from any intervention [30]. Given the novelty of such a program, we aim to understand the feasibility of this 12-week program in terms of usability and acceptance using a validated scale and attendance rates and explore measures of digitally collected physiologic improvement in adults aged 60 years and older.

## Methods

### Program Description

Our program, Strong Foundations, is drawn from several rigorously tested fall and fracture prevention exercise programs [11,31-33] but is new and entirely designed to be delivered digitally. This design emphasized exercises that were able to be remotely instructed, witnessed, and performed in a limited physical space such that most users would be able to use readily available household spaces. The program was designed with physician input and by exercise physiologists and a Doctor of Physical Therapy, all with extensive training in both group and individualized exercise for older adult populations. Strong Foundations is a 12-week iterative curricular program with 3 core components: postural alignment and control, balance and mobility, and muscular strength and power. All the exercises offered over the course of the intervention are designed to be appropriate for the target population (ie, older adults) and are standardized so all participants receive the same basic instruction, but the level of difficulty is scaled to participant experience, capability, and musculoskeletal limitations. This individualization was enabled by real-time delivery, such that instructors are able to adapt, respond, and tailor specific aspects of any class to fit the needs of participants, including independently pulling our participants into a “Breakout Room” for direct one-on-one instruction before reassimilating into the larger group. The 12 weeks of instruction establish and build such that throughout the program, posture and positions prioritized early in the program are used to build into more complicated maneuvers designed to improve the foundational techniques and activities that will allow participants to maximize learning opportunities and optimize gains related to balance, posture, and strength.

### Real-Time Feedback

Differentiating this program from other streamed and real-time web-based interventions available, the novel feature of this program is the delivery of semi-individualized instruction in real time within a small group setting. This is accomplished with a specifically trained “support” instructor providing guidance and correction on optimal exercise form while the “lead” instructor demonstrates for the larger group. This semitailored approach allows the lead instructor to provide “best practice” instruction and provide a visual demonstration while assistant instructors assure that participants are not only gaining important skills related to fall-prevention technique, but also doing this safely and remotely, as numerous screens on a digital interface would otherwise compromise the integrity of a single instructor. All instructors are aware of the curriculum in its entirety and versed on adaptive approaches for a participant who may not be able to do specific components safely or without predisposing to injury. An example of this may include someone with a known right shoulder injury, whereby certain resistance band and posture actions need to be modified—thereby the lead instructor may continue providing instruction to the group at large, with uninterrupted group synchronous interaction, but the support instructor can provide individualized recommendations to a specific participant without disrupting the overall dynamic.

### Program Development

The program was iteratively designed based on two, 4-week pilot classes with 9‐12 participants each (total n=21), which refined and informed the curriculum to inform the design of a final total 12-week program, where foundational components are expanded in intensity and challenge, assuring continual growth and improvement of participants. Participant experience was surveyed, with respondents (n=14) noting that the interface was easy to follow, with sufficient audio and visual interface for safely following instruction. Furthermore, 100% (14/14) of participants agreed or strongly agreed that “instruction was clear and easy to follow” and “I felt safe doing the exercises.” In addition, 86% (12/14) agreed or strongly agreed to “I could hear the instructors well” and “instruction was easy to see and follow.” In total, 64% (9/14) reported awareness of instructors watching validated tools to establish usability, however, were not collected in this early phase of development.

Final program development was designed to incorporate approximately 1 hour of instruction for 12 weeks with key aspects to establish the weekly focus, and materials were generated for dissemination between classes to participants to encourage and underscore the importance of ongoing practice to support gains during this intervention (Table 1).

**Table 1.** Strong Foundations curriculum focus across 12 weeks.

Week	Foundational exercise 1	Foundational exercise 2	Foundational exercise 3	Foundational exercise 4	Focus of the week
1	Neutral spine and marionette pose	Pelvic tucks and tilts	Hip hikes	Hip hinge	Posture
2	Quick feet drills	Review hip hinge	“Traditional abs”	Self-test: static balance	Balance
3	Front and lateral arm raises (with band)	Hip hinge (review) with squat and chair sit, stand	Reverse lunge	Heel raises	Strength
4	Neutral spine and pelvic tuck and tilt—standing	Static balance	Reverse lunge with progression	Heel raises with tennis ball	Combine 3 pillars
5	Sit and stand transitions	Around the clock steps	Hip hinge with choice of arm extensions	Modified and regular jumping jacks	Floor to stand transition—floor exercises
6	Hip hinge (review) with squat and chair sit, stand	Lunge with chair assistance progression	Single leg heel raises	Hing hinge + picking up objects	Compound strength movements
7	Drinking bird	Squat to lateral leg raise	Single leg heel raises	Toe raise walk around chair	Increase balance challenge
8	Side squat	Wall sit with wood chop	High knee walk	Quick feet multidirectional	Odd impact and multidirectional movements
9	Weight transfer with split stance	Squat into a High knee and arm reach	High knee walk	Head rotations in a tandem stance	Combine balance with compound movements
10	Single leg stance with band pull downs and serial 7’s	Lunge with chair support and head rotation	Standing superman with cognitive challenge	Goal post with arm slides	Introduce cognitive challenge
11	Reaching squats	Lateral jacks	Vertical push-ups	Knee drivers	Speed and power movements
12	Good morning	Bow and arrow	Hands up	Heel toe sitting with resistance band	Collaborative workout

### Site, Eligibility, and Enrollment

Participants were recruited through established venues from the Exercise and Physical Activity Research Center (EPARC), an exercise science–based laboratory with experience in physiologic phenotyping based on the University of California, San Diego campus. Given that the intervention was developed and deployed during the COVID-19 pandemic, all recruitment, enrollment, and measurement was done remotely; no face-to-face interaction or measurement occurred throughout the pilot. Given the novelty of the delivery platform and development of this program, our primary aims centered on determining its’ usability and feasibility in this context. Therefore, out of an abundance of caution and given the entirely remote recruitment, enrollment, delivery, and measurement through this program, this pilot included those with minimal-to-no-risk factors for falling based upon the CDC’s Staying Independent brochure questionnaire [34]; for this pilot, a participant was considered no more than a moderate fall risk based on a score of ≤4 [16]. Textbox 1 shows the eligibility criteria. Informed consent was performed remotely through secure digital methods (Docusign) with a real-time Zoom-based meeting with staff to review protocols and consent documents and establish an appropriate room setup. This included ensuring that there was sufficient space to move while also being at a sufficient distance from the camera that their entire body was visible to the instructor. In addition, before remote measurement, participants were individually coached on how to measure an appropriate length course (ie, 3 meters) and have an appropriately sized chair (ie, approximately 17-inch chair height without arms). Enrollment occurred from August to September 2021, with 39 older adults recruited and cohorted based on predetermined times of the intervention such that participants could select the program time most convenient for their schedule.

Textbox 1. **Eligibility criteria for Strong Foundations pilot.**

Inclusion criteria:

- Age 60 years or older and ambulatory, including with the use of a cane or walker (1 participant enrolled age 56, which was an unintended protocol deviation).
- Completion of the Stopping Elderly Accidents and Deaths Initiative (STEADI) Stay Independent Brochure.
- Access to internet and computer and a Zoom-interface or broadband.

Exclusion criteria:

- Individuals who are wheelchair bound.
- Exclusively communicate in a language other than English.
- Score of 4 or more on the STEADI modified questionnaire.

### Primary Feasibility Outcomes

Programmatic development and launch were aimed at determining feasibility and usability of a digital platform with an older adult population. Attendance was measured weekly as well as self-reported number of exercises performed outside of the class based on the previous week’s instruction and usability determined at completion of the program based on the System Usability Scale [35] (SUS), a nonpriority validated questionnaire, which was designed to understand the ease of use of new systems or programs using a 5-element Likert scale.

In general, scores >70 on the SUS are considered to have appropriate acceptability of a program or platform. The SUS was selected as the primary outcome, given it is considered the most widely used scale in eHealth to determine usability [36]. Finally, a survey to understand subjective experience was developed and deployed at the end of the intervention.

### Exploratory User Outcomes

To understand user experience and improvement, at baseline and at the completion of this curriculum, digitally collected objective measures of frailty and fall risk were collected to explore the opportunity to use the digital interface to collect these measures and understand changes from this intervention. All participants at baseline and after completing the intervention period (±1 week) were assessed remotely with the measures outlined and endorsed by national and international public health authorities, namely the timed up and go (TUG) [37-39] and 30-second chair rise [40]. Participants were then monitored and timed remotely while completing the assessments. TUG was completed with instruction to complete at both a normal speed (TUG normal) and “as fast as safely able to perform the test” (TUG-Fast). Given enrollment included those with few risk factors for falling, these physical measurements were selected because of their relative ease of administration and interpretability but remained exploratory from the lens of feasibility.

### Sample Size Determination

Power calculation was not conducted given the aim to determine feasibility and usability for this program and the selected outcome, the SUS, has been shown to be valid in small samples [41]. Therefore, we used an assumed attendance rate of 70%, which has held consistent in national programs in both rural and urban areas for older adults in fall prevention programs [42] and similar fall prevention programs that have evaluated this time frame to be sufficient to improve measures, markers of frailty, and fitness in different health contexts between 20 and 60 individuals [43-45]. Given budgetary limitations, our plan to enroll 4 cohorts all with at least 8‐10 older adults would be sufficient to determine both the feasibility and usability and explore physiologic changes.

### Statistical Analysis

Primary outcomes to understand feasibility and usability were determined based on the validated SUS [35], which in general assumes an average score of 68 and higher indicates more usability. Attendance rates were determined by a participant joining the Zoom interface measured weekly for each individual, reported as averages (mean) and SD. Numerical averages (mean) of patient-reported questionnaire results (out of 10) were calculated. Associations with SUS scores were calculated by Pearson correlation coefficients. Secondary outcomes were measured using paired-sample 2-taile*d t* tests for differences (for the TUG and 30-second chair rise, collected digitally) with corresponding mean differences and 95% CIs. Statistical analysis was performed on SPSS (version 28; IBM Corp).

### Ethical Considerations

This study was reviewed and approved by the University of California San Diego Institutional Review Board (#802148); informed consent was obtained by all participants. All study data were deidentified. Participants were not compensated to participate.

## Results

### Participants

Across 12 weeks, 38 of 39 (97.4%) recruited older adults completed the program. One participant left after 2 weeks of instruction because of a family emergency. Of the 38 who completed the program, the average age was 72 years (SD 5.5), and 37 were female (97.4%). Participants were spread across 4 cohorts, ranging in size from 9‐10 individuals.

### Feasibility and Usability

On average, participants attended 10 weekly sessions out of 12 (84%). The average number of home exercise sessions performed weekly between classes was 2.3 (SD 0.7). The average SUS score was 80.6 (SD 15.4), with a range of 40 to 100. On a 10-point scale, the average self-reported overall experience of the intervention and exercise classes was 8.5 (SD 1.9). No participants experienced an injury or had any safety related concerns, and additional subjective feedback was excellent with most participants eager for ongoing involvement and instruction. SUS scores were statistically inversely associated with age (Pearson correlation *r*=−0.411; *P*=.10) and positively with reported satisfaction score (Pearson correlation *r=*0.654; *P*=.004) but not statistically associated with attendance or improvements in TUG or 30s chair rise scores. Table 2 shows the number of participants and their SUS score range.

**Table 2.** System Usability Scale (SUS) score range.

SUS Score	Participants (n=38), n (%)
<50	2 (5)
50‐60	2 (5)
61‐70	6 (16)
71‐80	7 (18)
>80	21 (55)

### Objective Measures

Pre- and postmeasures were collected for 36 of the participants for the TUG and 35 of the participants for the 30-second chair rise. Baseline TUG at the “normal” speed was 8.8 (SD 1.9) seconds, and at the fastest possible speed was 6.2 (SD 1.4) seconds. Baseline 30-second chair stands were 14.5 (SD 3.4) stands. Post intervention, TUG and TUG-Fast were 8.0 seconds (SD 1.3 seconds; difference of 0.8 [95% CI 0.24-1.21] seconds) and 5.7 seconds, respectively (SD 1.1 seconds; difference of 0.48 [95% CI 0.22-0.75] seconds) and the 30 second chair rise increased to 18 stands (SD 4.1 stands; difference of –4.8 [95% CI –2.6 to –6.9] stands) (Table 3). At 12 weeks, 64% of all participants improved in the TUG (23/36, for both measures) and 91% (32/35) improved the chair stands. Reasons reported for being unable to complete these were varied but included participants traveling internationally, unrelated knee pain, and a single participant dropping as noted above after 2 weeks.

**Table 3.** Digitally collected objective markers of fall-risk reduction.

Measure	Baseline, mean (SD)	Post intervention, mean (SD)	Difference (95% CI)	Change (%)
TUG^a^ normal (seconds)	8.8 (1.9)	8.0 (1.3)	7.24 (0.24‐1.21)	9
TUG-Fast (seconds)	6.2 (1.4)	5.7 (1.1)	0.48 (0.22‐0.75)	8
30-second chair rise (stands)	14.5 (3.4)	18 (4.1)	−4.8 (−2.6 to 6.9)	24

a TUG: timed up and go.

## Discussion

The development and rationale behind the need for a digital curriculum, Strong Foundations, is described here. In this 12-week pilot, we found a high level of usability and feasibility of this program based on the high attendance rates, SUS scores, and self-reported participant experience. In addition, we found impressive changes in digitally collected measures of functional improvement in most participants, suggesting that the program increased strength and mobility and may reduce the risk of falls. Clinically, the 8%‐20% improvement in measurement after 12 weeks is notable, especially considering recruitment generally was limited to those with limited risk factors for falling and have higher levels of function and therefore less scope for improvement or gain. While exploratory in nature, these gains are promising to show improvements across this intervention even in a relatively lower risk group of older adults.

This 12-week fall prevention program was well accepted and showed promise related to reducing fall risk in an older adult population. The usability and safety (as evidenced by no adverse events) of the program indicates that digitally delivered real-time exercise instruction has potential in this population to reduce barriers and increase availability of fall prevention programs. While those enrolled were relatively low risk for falls (as evidenced by the STEADI score used for eligibility), results should be contextualized that even those who were generally doing reasonably well have opportunity to improve. Indeed, in other populations, such as older adults with hip osteoarthritis, a change in TUG of around 1 second and a 30-second chair stand of around 1 second and 2 stands, respectively as meaningful [46], and between 0.9s and 6.0s depending upon the quality of life assessment tool in patients who were postoperative [47]. While our results of TUG change of 0.8 seconds (normal) is less than these noted changes, given the health status of our enrolled participants and limited 12-week intervention, the improvements are still notable. Furthermore, our improvement of 3.5 stands in 30 seconds, especially from a baseline of 14.5 stands—a notable good baseline—shows the resilience and opportunity for improvement in lower extremity strength regardless of starting health status.

However, this study is not without limitations. This project recruited heavily from an environment with a relatively high socioeconomic status. As such, there may be a bias in enrolling individuals who already have digital tools like smartphones, tablets, and laptop computers and a high level of experience with digital technology, and therefore, a better understanding how to use these systems. As such, these participants may not be representative of the larger older adult population who may not have the same level of access to or understanding of such digital systems. We also did not ask specifically about their experience with previous technologies, though participants all did have sufficient equipment for enrollment. We also primarily enrolled women, which limits interpretability and generalizability of findings, especially given recent data suggesting higher fall-related impairment among older adult men [2]. Despite this, the use of a validated scale focused on usability commonly used across several domains related to eHealth and technology deployments [48], findings related to usability are supported; however SUS deployment in older adults has been challenged in some situations, namely those with cognitive impairment [49]. While we did not specifically screen for this in our enrollment, it is possible findings may be diluted. Furthermore, while the reasons remain unclear, the SUS average may be different and higher in digital physical activity interventions [48], making contextualizing results important; although, such data in older adult subpopulations remain limited. While we also did not only enroll participants who had experience with Zoom, it is possible that interested individuals may not have expressed willingness to enroll who were otherwise intimidated by such platforms, although we did not anecdotally experience this feedback. Subjective survey experience reports should be interpreted with this consideration [27]. Finally, it is not clear if digitally collected measures of physical performance, specifically the TUG and 30 second chair rise, are valid and reliable when compared with measures gathered in the clinic or laboratory. As such, the changes in physical performance demonstrated here, while promising, should be viewed as preliminary and not necessarily indicative of the same degree of change as would be expected were the data gathered in a more traditional context. However, given the rate of technology availability has continued to increase, attention is warranted to understand how these interfaces may be used for older adults [27].

In conclusion, this pilot showed that a digital interface is both usable and feasible but also suggested promise at improving functional measures associated with fall risk. Based on the increasing yearly number of falls and the relatively small number of participants who are engaging in fall-prevention programs, it seems clear that existing in-person exercise delivery modalities are insufficient. Strong Foundations offers a safe alternative to provide a real-time fall prevention program. Furthermore, the real-time feedback likely provides participants with added sense of purpose and value in their investment in improving their functional ability and therefore provide increased motivation for sustained behavior. Given the growing population and epidemiology of falls, having numerous options that enable engagement of older adults into high quality exercise programs designed to improve strength, postural alignment, and balance is increasingly important. Strong Foundations has promise to augment existing opportunities in this population and further leverages digital tools to increase the breadth of delivery potential. Ongoing attention and research is needed to both understand the scalability and sustainability of this program and to confirm that the observed changes in function and mobility through validated laboratory-based methods are replicated.

## Abbreviations

30 CS: 30-second chair stand
CDC: Centers for Disease Control and Prevention
EPARC: Exercise and Physical Activity Resource Center
STEADI: Stopping Elderly Accidents and Deaths Initiative
SUS: System Usability Scale
TUG: timed up and go
USPSTF: United States Preventive Services Task Force
